# Ridge Recontouring with Simultaneous Implant Placement Using Autogenous Bone Core Grafts

**DOI:** 10.3390/jcm14103541

**Published:** 2025-05-19

**Authors:** Horia Mihail Barbu, Andreea Sorina Petris, Stefania Andrada Iancu, Alexandru Burcea, Andreea Mariana Banateanu, Ana Caruntu

**Affiliations:** 1 Oral Implantology Department, Faculty of Dental Medicine, Titu Maiorescu University, 031593 Bucharest, Romania; horia.barbu@gmail.com; 2 European Centre of Oral Implantology, 011473 Bucharest, Romania; 3 Titu Maiorescu Doctoral School of Dental Medicine, 040441 Bucharest, Romania; 4 Postgraduate Residency Program in Oral Surgery, Faculty of Dental Medicine, Titu Maiorescu University, 031593 Bucharest, Romania; alexandru.burcea@helpdent.ro; 5 Department of Prosthodontics, Faculty of Dental Medicine, Titu Maiorescu University, 031593 Bucharest, Romania; andreea.banateanu@prof.utm.ro; 6 Department of Oral and Maxillofacial Surgery, “Carol Davila” Central Military Emergency Hospital, 010825 Bucharest, Romania; ana.caruntu@gmail.com; 7 Department of Oral and Maxillofacial Surgery, Faculty of Dental Medicine, Titu Maiorescu University, 031593 Bucharest, Romania

**Keywords:** bone regeneration, tissue engineering, reconstructive surgical procedures

## Abstract

**Background:** The autogenous bone core block (BCB) is a viable, biologically advantageous, and minimally invasive alternative to other augmentation procedures for small bone defects around dental implants. This study focused specifically on horizontal vestibular defects in the mandible, a frequently encountered yet underrepresented clinical situation, to evaluate the effectiveness and predictability of bone core grafting. **Methods:** Cylindrical autogenous bone cores, harvested from the implant-site osteotomy using trephine drills with a 2.5 internal diameter, were stabilized with osteosynthesis screws, and implants were placed simultaneously. Initial preoperative measurements of the edentulous ridge width were performed based on cone beam computer tomography (CBCT). At 4 months postoperatively, a subsequent CBCT measurement was performed for each implant site. **Results:** A total of 38 augmentation procedures were analyzed with a mean horizontal bone gain of 1.8 mm (*p* = 0.000). Improved outcomes were observed in V-shaped defects with remaining vertical bony walls, which contributed to better graft stability and volume preservation. While Khoury et al. previously validated the general applicability of this technique across various defect types, our study refines its indication by offering a clear protocol tailored to a common clinical niche. **Conclusions:** The proposed BCB method proved to be a safe, efficient, and with reduced morbidity procedure, providing clinicians with a practical and evidence-based tool for predictable horizontal bone augmentation.

## 1. Introduction

Treatment planning for dental implant insertion should follow prosthetic principles whenever possible [1]. Placing small-diameter implants in a residual alveolar ridge affected by mild bone loss may offer technical ease, but it can negatively impact the final prosthetic and aesthetic outcome, as well as the long-term success [1]. Therefore, the fundamental principles of implant-supported restorations, such as biologically favorable contours and biomechanical considerations including an implant-abutment design, torque, and cantilever configuration, play a significant role in implant survival [2,3]. In addition, both mobile and fixed prostheses must meet the patients’ functional and aesthetic expectations by positioning artificial crowns or dentures within the neutral zone, where muscular forces are balanced, contributing to comfort within the dento-maxillary system [4]. A key challenge in prosthetically driven implant placement is the quality and quantity of the residual alveolar bone [3,5]. In many cases, the edentulous crest is insufficient in width or height, due to periodontal disease, trauma, or iatrogenic triggers, and cannot support ideal implant positioning without prior or simultaneous grafting procedures [6]. Alveolar bone resorption remains the most common type of facial bone defect and has both functional and social implications. The need for bone augmentation in the context of implant placement was found to be high, with 63.68% of cases requiring an additional surgical technique [7]. Oral surgery procedures have advanced substantially both in terms of the augmentation materials and surgical techniques, succeeding in reduced patient morbidity, a decreased number of treatment stages, improved postoperative comfort, and superior long-term successful outcomes [8]. In cases of mild defects of the alveolar bone, it is possible to consider single-stage procedures of bone augmentation and implant placement, using autologous bone—the gold standard in bone regenerative procedures—or using other types of biomaterials. A more recent approach involves the use of autologous bone cores harvested from the implant osteotomy site, as first described by Fouad Khoury [9]. The main advantage of this technique is the use of autogenous material with its osteogenic capacity and biologic compatibility with the recipient surgical site [9]. The method enables single-stage implant placement and simultaneous alveolar bone reconstruction with autogenous bone. The method is granted as simple, safe, and efficient for the reconstruction of limited bone defects [9].

Compared to the research of Fouad Khoury, the rationale of this study is to evaluate the outcome of this technique exclusively for vestibular augmentation in the mandible. This research is meant to bring to light the feasibility of horizontal augmentation surgeries with a bone core harvested from the implant site, after previous bone grafting, or just as single grafting procedures.

## 2. Materials and Methods

### 2.1. Study Design

The research was focused on 38 surgeries, which combined implant placement with bone core grafting procedures for minimum atrophies of the edentulous crest. The initial and postoperative widths were measured to point out the efficacy of the bone augmentation technique in the lower jaw. By focusing exclusively on vestibular augmentation in the mandible, our study provides a more targeted and controlled analysis of localized bone gain, assessing the efficacy of BCB in a specific and well-defined clinical setting. The study design followed the criteria of a retrospective cohort analysis, respecting the STROBE (Strengthening the Reporting of Observational Studies in Epidemiology) guidelines [10]. The entire research was performed in Prof. Dr. Barbu Clinic, located in Bucharest, Romania. The database included intraoperative pictures of the clinical cases, cone-beam computed tomographies (CBCTs) before and after bone grafting procedures, and the medical records of the patients, with detailed descriptions of the procedures and specific determinants of each individual. The entire analysis respected the ethical principles of the Declaration of Helsinki (2008) and the following amendments (Fortaleza 2013) concerning studies implemented on human patients. The research was conducted with the approval of the Ethics Committee of Titu Maiorescu University, Bucharest No. 1/03.13.2024 at the beginning of this research.

### 2.2. Study Population

All participants agreed to take part in this research and provided signed informed consent. They were all eligible for oral surgeries, without any health conditions or medications that would contraindicate elective surgical procedures or could interfere with the wound healing or the osseointegration of the implant or bone graft (e.g., uncontrolled systemic disorders, oncological diseases under treatment, radiotherapy in the neck and head area, bisphosphonate therapy). The inclusion criteria related to the alveolar bone status were as follows: partial edentulism, alveolar bone thickness of a minimum of 5 mm, dehiscence or fenestration around the implant neck at insertion, adequate primary stability of the implant, medium-to-hard bone density (D2 or D3 according to the Misch classification, corresponding to 1250 HU (Hounsfield units) up to 350 HU, respectively [11]). The exclusion criteria were a bone thickness less than 5 mm, low bone density (D4, corresponding to <350 HU [11]), decompensated general conditions, and pregnancy.

### 2.3. Surgical Protocol

**Figure 1:**
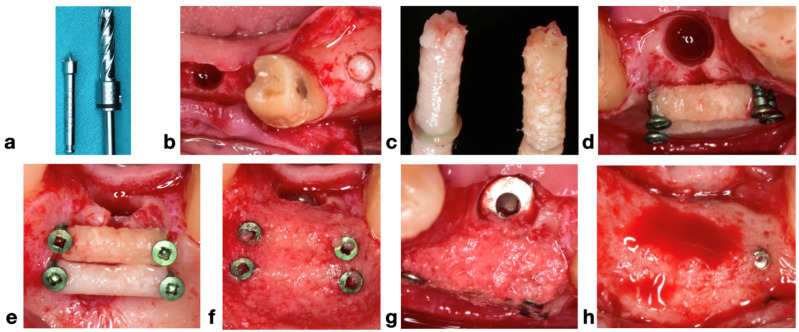
(**a**) The trephine pilot drill and the corresponding trephine drill; (**b**) the outlining of the future block and the osteotomy; (**c**) bone core blocks; (**d**) bone block fixation with the aim of screw heads; (**e**) lateral view of fixed bone blocks; (**f**) autologous chip augmentation; (**g**) implant insertion; (**h**) result after 4 months.

Based on a preoperative CBCT analysis, eligible patients were informed about the treatment plan and, after signing the informed written consent, were scheduled for the procedure (BCB). Each patient received a preoperative regimen consisting of 2 g of amoxicillin/clavulanic acid (875 mg/125 mg per tablet) and a single 4 mg dose of dexamethasone sodium, administered one hour before the surgery. Each procedure was initiated with infiltration with local anesthesia: articaine with epinephrine 1:100,000 (Ubistesin Forte, 3M ESPE, Seefeld, Germany). The crestal incision with a vertical-releasing incision was performed with a 15c blade. The one-stage minimally invasive approach was performed by harvesting a cylindrical bone block from the osteotomy site using the Trephine ejection kit (Meisinger, Neuss, Germany). The outline of the future bone block was made using a designed trephine pilot drill (2.5 internal diameter/3.5 external diameter) from the kit, which helps the next trephine drill to engage directly into the bone, avoiding the sliding that usually occurs. Next, the corresponding trephine drill is inserted with full speed (800 rpm) into the shank created by the pilot drill to the desired depth (6–8 mm). The bone core length should have a minimum length of 6 mm for a 4 mm-diameter implant. This trephine drill was intermittently slid into the bone, smoothly, hand-controlled, using high speed with copious irrigation. After the desired depth is reached, the drill is removed carefully, preventing the fracture of the bone core. In some cases, the bone core remains inside the residual crest and in others into the trephine drill. The bone core is carefully removed with instruments, specially designed for removing it from the crest or from the drill. After the block is collected, it will be placed in saline. The implant site preparation is carried out with the drills from the implant surgical kit until the desired depth of the osteotomy is reached. The drilling will be biologically driven, with low speed (maximum 50 rpm), no irrigation, and careful suction, trying to harvest as much autogenous bone chips as possible. Figure 1 presents the sequence of BCB.

The block is then fixed on the buccal crest in such a manner that the osteosynthesis screws will not interfere with the implant site. The bone block fixation technique is modified compared to the one described by Fouad Khoury: the first screw is placed mesial to the implant site, perpendicular to the buccal plate, using a 0.8 mm drill and a 7 mm length with 1 mm-diameter screw from (Devemed GmbH, Tuttlingen, Germany). After the screw is inserted partially, the block is positioned in contact with it, and then, while holding the block with forceps, the second hole is made while the drill is in contact with the block. The second screw is then inserted, and alternatively, both screws will be inserted until the screw heads press the autologous bone block to the buccal wall. After the block is fixed, the implant is inserted, and the rest of the harvested bone chips are placed above and around the bone block. The flap is repositioned and sutured without tension using horizontal mattress sutures.

Implant insertion was performed in the same surgical stage, with an aimed primary stability < 30 Ncm. Prosthetic loading was postponed after 4 months, to allow for the implant and bone graft integration. All patients were included in the standard postoperative monitoring protocol for implant-based dental rehabilitation with visits at 7 days, 1 month, and 4 months after surgery and every 6 months after implant loading.

The surgical sites were analyzed based on CBCT performed 4 months postoperatively. All tomographies were performed with the same device having a self-calibration method included.

### 2.4. Statistical *Analysis*

Statistical analysis was performed using IBM SPSS Statistics, version 20. The major point of interest was the subsequent bone gain after grafting, patient characteristics, and implant success rates. Statistical data were reported as the mean value, plus the standard deviation (SD). A Shapiro–Wilk test was used to determine the normality of distribution. The Spearman rank-order correlation test was selected to analyze the association between the study variables, considering its robust character in the context of a small sample size. For homogeneously distributed data, the analysis was conducted using the independent *t*-test. Statistical significance was confirmed for a *p*-value below 0.05.

## 3. Results

### Study Group Analysis

**Figure 2:**
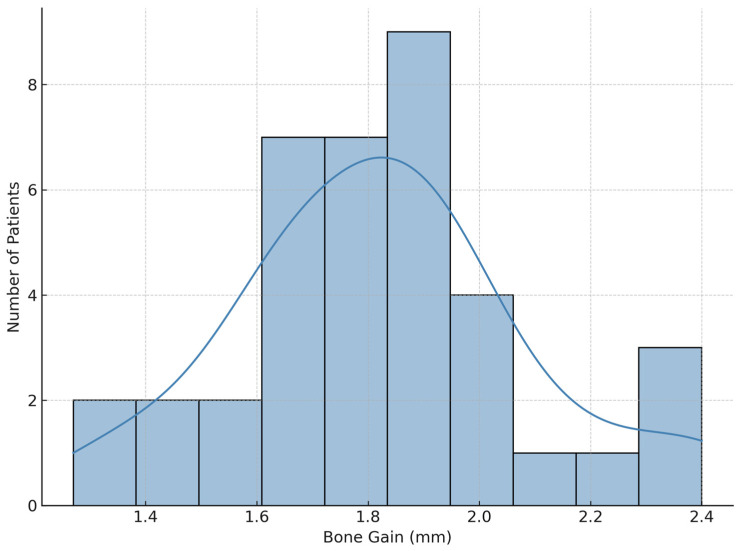
Bone augmentation using BCB technique four months after surgery: mean horizontal bone gain of 1.81 mm (SD = 0.28), with a range of 1.27 to 2.4 mm (*p* = 0.000).

The study included 38 surgical procedures of horizontal bone grafting techniques using small cylindrical bone blocks, consisting of bone cores harvested directly from the implant osteotomy site. There were 29 female (76%) and 9 male patients (24%), with a mean age of 49.11 years old (SD = 12.27), ranging between 26 and 77 years old. The group of patients included 32 nonsmokers (84%) and 6 smokers (16%), reporting a daily consumption of more than 10 cigarettes (Table 1). All surgeries were performed in the mandible, as a single augmentation procedure using mini bone blocks with a diameter graft of 2.5 mm harvested from the implant bed alone and with simultaneous implant placement. The morphology of the bone defects was classified into two groups: V-shaped defects with vertical walls in 23 cases (60.5%) and horizontal defects without vertical walls in 15 cases (39.5%). The initial width of the alveolar crest had a mean value of 5.95 mm (SD = 0.72),ranging from 4.8 mm to 7.61 mm. A total of 90 osteosynthesis screws were used to fix the core bone block around implants of different brands, lengths, and diameters. Out of the 90 screws, 72 were removed after 4 months, during implant uncovering stage. Improper implant integration was encountered in 1 case (2.6%) observed in the first month postoperatively. The implant was removed without any damaging the graft, which remained stable and fully integrated. A new implant was successfully inserted at the same site after 2.5 months. In the remaining 37 cases (97.4%), stable integration of both the implant and graft was confirmed at reentry. The mean horizontal bone gain measured on CBCT scans 4 months postoperatively was 1.81 mm (SD = 0.28), ranging between 1.27–2.4 mm, representing a significant increase compared to baseline (*p* = 0.000). Figure 2 illustrates the distribution of postoperative bone gain (in mm) and its frequency within the study group.

**Figure 3:**
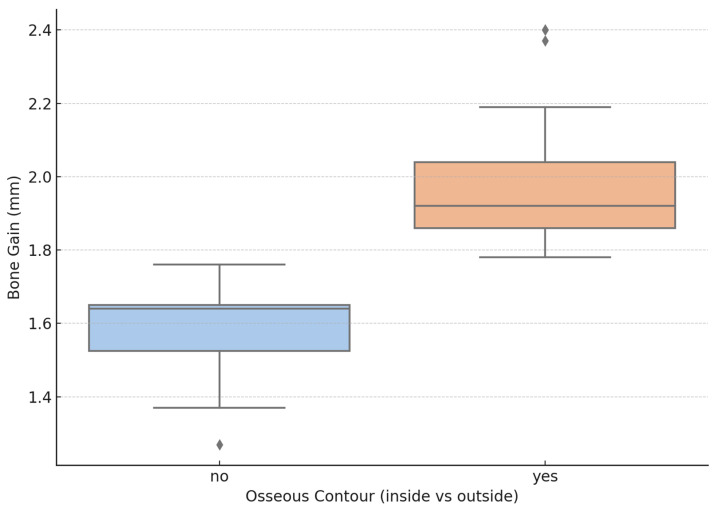
Bone augmentation depending on defect type: greater bone gain was achieved when the block was placed within a V-shaped defect with vertical walls (orange), having a mean value of 1.98 mm (SD = 0.19), compared to 1.58 mm (SD = 0.14) in defects without osseous contour (blue).

Greater bone gain was observed when the block was positioned inside a V-shaped defect with vertical walls, with a reported mean value of 1.98 (SD 0.19 mm), compared to defects lacking a bony containment, where the mean gain in volume was 1.58 (SD = 0.14 mm). The difference between the two types of defects showed a strong statistical significance (*p* = 0.000), the two groups being comparable in terms of baseline ridge width (*p* = 0.447). These findings highlight the importance of defect morphology in determining the outcome. A visual representation of the discrepancy between the outcomes for the two types of bone defects is shown in Figure 3.

Based on the Spearman test, a significant and very strong association was found between horizontal bone gain and the variable representing the positioning of the bone core block relative to the native osseous contour (r = 0.835, *p* < 0.001), with a determination coefficient of 0.69. This was further supported by independent *t*-test comparison (t(36) = 7.159, *p* < 0.001), confirming that the morphology of the defect, with containing vertical walls is beneficial for the subsequent bone gain after augmentation with core bone blocks. In addition, a statistically significant correlation was observed between bone gain and patient sex (r = 0.33, *p* = 0.043), suggesting that gender may exert a minor yet measurable influence on the regenerative response, in favor for female patients. Conversely, no significant correlations were found between horizontal gain and other clinical variables such as age (r = 0.006, *p* = 973) or smoking status (r = 0.198, *p* = 0.234). These findings suggest that the technique’s success is primarily dependent on defect morphology and graft positioning rather than patient-related systemic factors.

At 4 months postoperatively (6.5 months in the case of implant replacement), all implants were successfully loaded with screw-retained crowns. All patients underwent biannual clinical and radiographic evaluations, with a minimum follow-up of 1.8 years and a maximum of 8.3 years postoperatively. There were no signs of inflammation, the periimplant soft tissue had a normal, healthy color and texture, while panoramic radiological examination showed a stable bone contour around the implants in all patients. No other types of complications were encountered during the follow-up period.

## 4. Discussion

Without a harvesting technique, the bone volume at the implant site would otherwise be lost [12]. The BCB technique offers the possibility to harvest a considerable quantity of autologous grafting material—the gold standard in bone augmentation—directly from the implant site, reducing the morbidity of the surgery. Typically, augmentation procedures imply a second, donor site to harvest the required amount of autogenous material [12,13]. Other materials (xenograft, alloplast) which reduce the surgical morbidity because of their external origin, without a need of a donor bed, have less biological contribution to osseointegration processes [14]. An additional donor bed is another potential source of postoperative pain or other types of complications [13]. Trephine drills are widely recognized for their safety and efficiency in harvesting autologous cores from implant site. The ratio between compact/cancellous bone of the harvested bone block is different in previous grafted vs. non-grafted surgical sites [15]. A recent study from 2022 compared the thickness of the cortical plate on the top of the edentulous crest. The results revealed differences in anterior and posterior areas of the arch, maxilla versus mandible, or in grafted versus non-grafted sites [15]. Based on this imaging study from 110 mandible donor sites, the best quality bone with optimal cortical/cancellous ratio can be harvested from non-grafted mandibular posterior sites. Thus, drilling in native bone, without prior bone addition procedures, is optimal for the final bone augmentation results, while the ratio of compact to trabecular bone will depend on the depth of the implant site and will always be in favor of the cancellous bone [9,12]. From this point of view, bone core block grafting material includes the essential components for further vascularization and osteointegration [9,12,16,17].

While a greater amount of cancellous bone benefits the osseointegration process, low-density bone with rare trabeculae will not allow to harvest an appropriate bone cylinder from the donor site. This is the main reason why the core block technique is indicated in medium to hard bone types. Furthermore, the soft trabecular bone makes the core block difficult to stabilize with the osteosynthesis screws, or it can lead to block fracture during harvesting and fixation onto the recipient site. From another point of view, thick, avascular cortical bone may lead to greater graft resorption, hindering proper neoangiogenesis. When more than one implant will be placed, multiple core blocks can be harvested. Depending on the bone defect, the grafted blocks can be positioned on the edentulous crest, on the buccal plate next to each implant neck, or, in larger vertical defects, one bone block on the top of the other one. Our findings show that best results are achieved in the reconstruction of V-shaped bony defects of the recipient site. The findings support the hypothesis that bone defects with remaining vertical walls provide better graft stability, reducing resorption and leading to greater bone augmentation. Comparing other studies on the same surgical augmentation procedure, Fouad Khoury presented successful outcomes both for the mandible and for the upper jaw [9]. The author proposes this augmentation procedures as a secondary option, after previous bone grafting procedures had suboptimal results, due to partial bone graft resorption. Bone grafting surgeries for major atrophies are usually performed in two stages, with the bone augmentation alone in one stage and implant insertion subsequently. Minimal loss of the grafting material may be counterbalanced in the second surgery, with implant insertion simultaneously with core bone block augmentation [9,12]. There are some disadvantages of this augmentation technique: the complexity in manipulating the bone block during the harvesting process and its rigid fixation, especially due to the small dimension of the graft, the fracture of the block or, in some cases, the difficulty to remove the block from the donor site or from the drill. However, this technique also presents several notable advantages, including a relatively low learning curve that allows it to be mastered without requiring extensive surgical experience, the absence of a secondary donor site, and a reduced surgical time compared to other minimally bone grafting techniques.

The limitations of our study are related to its retrospective nature and the relatively small sample size. The potential bias was minimized by performing CBCT measurements by two distinct operators (S.A.I. and A.S.P.). To confirm our preliminary findings, future prospective, randomizes studies, involving larger groups of patients should be implemented.

Within the limitations of this study, the core bone block technique has proved to have predictable results in cases with minor bone atrophy, being a minimally invasive augmentation procedures, providing stable, qualitative augmentation, with minimal risk of complications and reduced overall costs. Although the results are promising, the technique has the limitation of being suitable only for the reconstruction of small bone defects.

## 5. Conclusions

The core bone block technique is a great choice in correcting minimal defects around implants. This procedure brings the biological advantage of saving the patient’s own material when creating the implant site, aiming for an increased bone volume around the implant, while avoiding the risks of an additional donor site.

## Figures and Tables

**Table 1:** Demographic and clinical characteristics of the patients enrolled in the study.

Total number of surgical procedures	38
Gender distribution	29 females
	10 males
Age range (years old)	26–77
Mean age (years old)	49 (±12.27)
Smoking status	32 nonsmokers (83.2%)
	6 smokers (15.8%)
Total number of osteosynthesis screws used	90
Total number of osteosynthesis screws removed	21
Implant diameter range (mm)	3.1 to 4.8
Implant failure	1 case (2.6%)
Mean initial width of the crest (mm)	5.95 (±0.72)
Bone graft width (mm)	2.5 (bone core diameter)
Average horizontal bone gain (mm)	1.8 (±0.28)
Follow-up period	1.8–8.3 years

## Data Availability

Data presented in this article is unavailable due to privacy reasons.

## Abbreviations

The following abbreviations are used in this manuscript:

BCB	Bone core block
CBCT	Cone beam computer tomography
SD	Standard deviation
